# Supplementary material for: MICFuzzy: A maximal information content based fuzzy approach for reconstructing genetic networks
Source: PLoS One. 2023 Jul 7;18(7):e0288174. doi: 10.1371/journal.pone.0288174 (PMC10328247; doi:10.1371/journal.pone.0288174)
Supplement: S1 Fig — (PDF) [file pone.0288174.s001.pdf]

Pseudo-code - MICFuzzy

**Algorithm**	MICFuzzy(Y, S)
**Input :**	Y : *Time series gene expression data of target gene, has T time samples, $Y=\{y_1, y_2,...y_i,..., y_T\}$ and y_i : expression value of target gene at i time point*<br>$S = \{X_1, X_2, .., X_j, .., X_n\}$: *Set of time series gene expression data in which each input gene X has T time samples, $X_j=\{x_1, x_2,...x_i,..., x_T\}$ and x_i : expression value of gene X_j at i time point.*
**Output :**	R : *Set of selected activator-repressor pairs*

```
1   R ← ∅ : Array of selected regulatory gene pairs
2   RS ← ∅ : Array of residual score values with corresponding gene pairs
3   NRS ← ∅ : Array of normalized residual score values with corresponding gene pairs
4   M ← ∅ : Selected gene list (X̄) from ITBM with evaluated MIC value
5   M ← ITBM(S, Y, δ), where δ : MIC threshold, M{X̄, MICValue}
6   For j ← 1 to |M| Do
7       activator = X̄_j ϵ M{X̄(X̄_1, ...., X̄_j , X̄_|M|)} : Setting activator gene
8       For k ← 1 to |M-1| Do
9           If X_k ≠ activator then
                Setting repressor gene, where X̄_k ≠ X̄_j and X̄_k ϵ M{X̄(X̄_1, ...., X̄_|M−1|)}
10              repressor = X̄_k
11              P ← ∅ : Array of predicted target gene expression levels (ŷ_t) of each time point
12              Rule ← ∅ : Array of number of times each rule fired by considered activator-repressor pair
13              For t ← 1 to T Do : T is the number of time points in the dataset
14                  rules ← ∅
15                  Reg_Effect_Act_t = M{MICValue(X̄_j, Y)} ∗ x_j,t
16                  Reg_Effect_Rep_t = M{MICValue(X̄_k, Y)} ∗ x_k,t
17                  ŷ_t , rules_t = Fuzzy_Model(Reg_Effect_Act_t, Reg_Effect_Rep_t, y_t)
18                  P ← P ∪ {ŷ_t}
19                  For h ← 1 to |Rule| Do
                        increment the values of each element of the Rule array
20                      Rule ← ({Rule}_h + {rules_t}_h) :
21                  End For
22              End For
23              MSE = EvaluateMSE(P, Y) : Evaluating mean squared error
24              Variance = EvaluateVariance(Rule) : Evaluating variance of values in array Rule
25              ResS = MSE * Variance : Evaluating Residual score
26              RS ← RS ∪ {ResS, (activator, repressor)}
27          End If
28      End For
29  End For
30  NRS ← Normalization(RS{RS}) : Normalizing Residual Score to [0-1]
31  If NRS{RS} < δ then : If normalized residual score value is < δ: The defined threshold of normalized residual score
32      R ← R ∪ {NRS{(activator, repressor)}} : Selecting the relevant pair of genes for target gene
33  End If
34  Return R
```

Line 5 – The gene expression values of the input gene set (*S*) and the given target gene (*Y*) are the input parameters of the *ITBM* method. MIC value or pair-wise association between each input gene and the target gene is evaluated in *ITBM* method. Since we disregard the self-regulations the target gene is excluded from the input gene set (*S*). The input genes with MIC values above the chosen threshold (δ) are selected as most pertinent input genes for the target gene. The selected gene list ($\bar{X}$) and their corresponding MIC values are the output ($M(\bar{X}, MICValue(\bar{X}, Y))$) of *ITBM* method. This method was implemented using *minepy* Python library (https://minepy.readthedocs.io/en/latest/).

Line 7 – A gene ($\bar{X}_j$) from the selected gene list, $M\{\bar{X}(\bar{X}_1, \ldots., \bar{X}_{|M|})\}$ is set as the activator.

Line 10 - A gene is set as a repressor from the selected gene list where the repressor is not the same gene as the activator ($\bar{X}_k \neq \bar{X}_j$ and $\bar{X}_k \in M\{\bar{X}(\bar{X}_1, \ldots., \bar{X}_{|M-1|})\}$).

Line 12 - *Rule* is an array that represents how many times each rule is fired by the considered activator-repressor pair. The size of this array (|*Rule*|) is the number of rules in the designed fuzzy control system. For example, the first element of this array contains the total number of times the first rule was executed by the activator-repressor gene pair across all time points (*T*) in the given dataset.

Line 15 – Evaluating the regulatory effect between the activator gene and the target gene at the considered time point(*t*).

*Regulatory effect of Activator(t) = MIC between activator gene and the target gene * gene expression level(t)*

Line 16 - Evaluating the regulatory effect between the repressor gene and the target gene at considered time point(*t*).

*Regulatory effect of Repressor(t) = MIC between repressor gene and the target gene * gene expression level(t)*

Line 17 – Execution of the Fuzzy model. The regulatory effect of the activator and repressor, and the gene expression value of the considered target gene at the considered time point (*t*) are the inputs to the *Fuzzy_Model* method. This method includes fuzzy control system which is implemented based on *Scikit-fuzzy* Python library (https://pythonhosted.org/scikit-fuzzy/overview.html). The original source code of the fuzzy control system is available in (https://github.com/scikit-fuzzy/scikit-fuzzy) and according to our requirements the original code can be modified. For example, the number of levels of input parameters and output parameters, and the number of rules can be changed according to the underline design of the fuzzy model. This method returns the predicted gene expression value ($\hat{y}_t$) of the target gene for a given time point (*t*) and array ($rules_t$) of the number of times each fuzzy rule is fired to generate this prediction at considered time point (*t*).

Line 20 – Each element of *Rule* array is incremented by the obtained values corresponding to the considered time point ($rules_t$: The number of times each rule (*h*) is fired by the considered pair to predict the target gene expression value at *t* time point).

Line 30 – Using min-max normalization evaluated residual scores in RS array are normalized into 0-1 range.

Line 31 & 32 – If normalized residual score of the activator-repressor pair is less than the defined threshold (δ), this pair will be selected as a relevant gene pair to the considered target gene and can be added to the array of selected activator-repressor pairs (*R*) of the given target gene.
